# Misoprostol vaginal insert versus misoprostol vaginal tablets for the induction of labour: a cohort study

**DOI:** 10.1186/s12884-018-1788-z

**Published:** 2018-05-10

**Authors:** Daniele Bolla, Saskia Vanessa Weissleder, Anda-Petronela Radan, Maria Luisa Gasparri, Luigi Raio, Martin Müller, Daniel Surbek

**Affiliations:** 1 Department of Obstetrics and Gynaecology, Inselspital, Bern University Hospital, University of Bern, Effingerstrasse 102, CH-3010 Bern, Switzerland; 2 Departments of Obstetrics, Gynaecology and Reproductive Sciences, Yale University School of Medicine, New Haven, USA

**Keywords:** Misoprostol vaginal insert, Misoprostol vaginal tablets, Induction of labour, Tachysytole, Misoprostol, Caesarean section, Neonatal outcomes

## Abstract

**Background:**

Misoprostol vaginal insert for induction of labor has been recently reported to be superior to dinoprostone vaginal insert in a phase III trial, but has never been compared to vaginal misoprostol in another galenic form. The aim of this study was to compare misoprostol vaginal insert (MVI) with misoprostol vaginal tablets (MVT) for induction of labor in term pregnancies.

**Methods:**

In this retrospective cohort study we compared 200 consecutive women induced with 200-μg misoprostol 24-h vaginal insert (Misodel®) with a historical control of 200 women induced with Misoprostol 25-μg vaginal tablets (Cytotec®) every 4-6 h. Main outcomes variables included induction-to-delivery interval, vaginal delivery within 24-h, incidence of tachysystole, mode of delivery, and neonatal outcome. A subanalysis in the MVI group was performed in order to identify predictive factors for tachysistole and vaginal delivery within 24 h.

**Results:**

The time from induction to vaginal delivery was 1048 ± 814 min in the MVI group and 1510 ± 1043 min in the MVT group (*p* < 0.001). Vaginal delivery within 24-h occurred in 127 (63.5%) patients of the MVI group and in 110 (55%) patients of the MVT group (*p* < 0.001). Tachysystole was more common in the MVI group (36% vs. 18%; *p* < 0.001). However, no significant predictors of uterine tachysystole in MVI group have been identified in crude and fully adjusted logistic regression models. Bishop score was the only predictor for vaginal delivery within 24 h (*p* < 0.001) in MVI group. Caesarean delivery rate (27% vs. 20%) and vaginal-operative deliveries (15.5% vs. 15.5%) did not differ significantly between the two groups. Neonatal outcomes were similar in both groups.

**Conclusions:**

MVI achieves a more vaginal delivery rate within 24 h and Tachysystole events compared to MVT. However, no differences in caesarean section, operative vaginal delivery, and neonatal outcomes are reported. No predictors of tachysistole after MVI administration have been identified. Bishop score and parity are the only predictors of vaginal delivery within 24 h after MVI administration.

## Background

Labour induction is a commonly performed procedure in obstetrics with an increasing incidence of approximately 25% [1–3]. In the latest decades, several pharmacological and mechanical methods of labour induction have been developed. Success of labour induction is linked to the Bishop score. An unfavourable cervix characterized by low cervical Bishop score decreases the success of labour induction and therefore is associated with a higher incidence of caesarean sections (CS) [4–7]. In this context, the use of prostaglandins has proven to be more effective for cervical ripening in women with low Bishop score as compared to other commonly used methods (oxytocin, Foley catheter, amniotomy), but is associated with an increased rate of uterine tachysystole, hyperstimulation syndrome, and uterine rupture [8, 9].

Misoprostol is a prostaglandin E_1_ analogue currently marketed as oral tablets for the prophylaxis and treatment of peptic ulcer disease. Although the obstetrical use of Misoprostol is off-label in most countries, an extensive literature have proven its safety, efficacy, and dose-response effect in labour induction at term pregnancies [8]. Its pharmacological characteristics compared to prostaglandins E2, along with its easiness of storage led to the widespread use in obstetrics [10, 11]. Moreover, the World Health Organization entered Misoprostol in the list of the essential drugs for obstetrical use and medical organisations such as the International Federation of Gynaecology and Obstetrics and the American College of Obstetrician and Gynaecologists recommended their use in pregnant women [2, 12–15].

In 2014 misoprostol was registered in Europe in form of a single controlled-release vaginal insert containing 200 μg, and approved for labour induction beyond 37 0/7 weeks’ gestation [16]. A large phase III registration trial reported a favourable outcome with a similar rate in vaginal deliveries and CS in comparison with dinoprostone vaginal insert [17]. Misoprostol vaginal insert (MVI) use resulted in a reduced induction-to-delivery interval, reduced time to active labour, and decreased need for additional oxytocin. At the same time, uterine tachysystole requiring intervention was increased (13.3% vs. 4%) [17], whereas no difference in neonatal outcome could be observed. So far, no data are available about the comparison between MVI and MVT. The aim of the following study was therefore to compare MVI to MVT in terms of vaginal delivery within 24 h and maternal/fetal outcomes. Secondary outcome was the identification of predictors of vaginal birth within 24 h. Furthermore, this study aims to identify the predictive factors for the occurrence of uterine tachysystole associated with MVI use, since it is the only significant adverse outcome reported in the MVI group.

## Methods

Between January 2012 and July 2016, a retrospective cohort study was conducted at the Department of Obtetrics and Gynaecology, University Hospital Bern – Inselspital (Switzerland). We included all consecutive women who had a labour induction > 36 0/7 weeks’ gestation. Before May 2014, MVT was routinely used off-label for labour induction in this patient population. In May 2014, MVT was replaced with the novel, approved MVI. The analysis periods were set as follows: January 2012 to 30 April 2014 for the MVT cohort and 1 May 2014 to 31 July 2016 for the MVI cohort. Data were obtained from the patients’ electronic medical records. Each patient signed an informed consent regarding data collection for scientific purpose. Exclusion criteria consisted in foetal malpresentation, previous CS or uterine scarring (e.g., previous caesarean section), < 36 + 0 weeks of gestation, premature rupture of the membranes less than 24-h before starting the induction, severe preeclampsia, body mass index (BMI) > 50, signs of maternal infections in peripheral blood samples, abnormal foetal heart rate tracings or signs of active labour at admission, and twin pregnancy. Patients received MVI (Misodel^®^, Ferring Inc., Saint-Prex, Switzerland) containing 200 μg misoprostol in a slow-release vaginal insert as a single application, left in place for a maximum of 24 h, or MVT with repetitive dosing every 4 h as indicated. MVT were prepared in the hospital’s pharmacy by crushing Cytotec^®^ (Pfizer Inc., New York, US) tablets containing 200 μg misoprostol and manufacturing custom-made vaginal tablets each containing 25 μg misoprostol. In each group, preparations were placed in the posterior vaginal fornix. Criteria for removing MVI and ceasing MVT administration were the onset of three or more contractions within 10 min, lasting for 45 s or longer, resulting in cervical change or leading to a cervical dilatation of 4 cm or more with any frequency of contractions, or after completion of the 24-h dosage period. If spontaneous rupture of the membranes occurred, antibiotic prophylaxis was started after 24 h or immediately if a vaginal group B streptococcal smear test was positive. In both groups, an interval of at least 30 min was set between the removal of the vaginal insert or the last administration of vaginal tablet and the start of intravenous oxytocin administration.

Baseline demographic data and patients’ characteristics were prospectively collected including maternal age, BMI, parity, contractions and membrane rupture status, gestational age, Bishop score (evaluated at the time of labour induction), and ethnicity. Each patient underwent at least 30 min of cardiotocography assessment to record the foetal status and confirm that there was no uterine pattern of active labour before the induction. Time and mode of delivery (vaginal, CS, operative vaginal) were recorded. Primary outcome was the rate of vaginal delivery within 24 h. Secondary outcomes were the induction-to-vaginal delivery interval (IDI), rate of caesarean section and operative vaginal delivery, the proportion of women requiring predelivery oxytocin, and the rate of uterine tachysystole. Further outcomes included the rate of peridural anaesthesia or other pain relievers, uterine hyperstimulation syndrome, postpartaum haemorrhage, uterine rupture, and length of hospital stay (days). Uterine tachysystole was defined by any occurrence of five or more contractions within 10 min, averaged over three consecutive 10-min periods [18]. Uterine hyperstimulation syndrome was defined as uterine tachysytole with concurrent foetal heart rate decelerations or bradycardia [18]. Neonatal outcome included the rates of 5-min Apgar score < 7, umbilical artery/venous pH, and umbilical artery base excess − 12 mmol/L, presence of meconium and transfer to neonatal intensive care unit. Ethical approval for the study was obtained by the local institutional review board (Ethics Committee of the Canton of Bern, Switzerland).

### Statistical analysis

The patients characteristics and the delivery outcomes of the two groups were compared using Student’s t-test for continuous variables and chi-square test for dichotomous variables. Continuous values were expressed as mean ± standard deviation. Unpaired continuous data were analyzed using the Mann-Whitney test. Proportions were compared by the Fisher’s exact test. A *p* value < 0.05 was considered to be statistically significant. *P* values with more then 3 decimals were reported as >/< 0.001. Age of the mother, BMI, ethnicity, parity, gestational age at delivery, Bishop score, indication for labour induction, and fetal weight were evaluated as predictors for vaginal delivery within 24 h and for tachysistole, in crude and fully logistic regression models (OR 95%). Data analysis was performed with GraphPad Prism version 5 for Mac (GraphPad Software, San Diego CA).

## Results

**Fig. 1:**
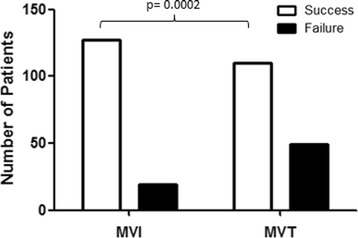
Vaginal delivery within 24-h after induction of labour with MVI compared to MVT. Abbreviations: MVI = Misoprostol vaginal insert, MVT = Misoprostol vaginal tablets

During the study interval a total of 400 women were included, 200 consecutive women induced with MVI and 200 consecutive women induced with MVT. The clinical characteristics of the study population are summarized in Table 1. Both groups were homogenous with similar baseline characteristics, except for BMI. Vaginal delivery within 24 h occurred in 127 (63.5%) patients of the MVI group and 110 (55%) patients of the MVT group (*p* < 0.001). Induction-to-vaginal delivery interval was 1048 ± 814 min and 1510 ± 1043 min in the MVI and MVT group, respectively (*p* < 0.001) (Fig. 1). Uterine tachysystole was more frequent in the MVI group (36% *n* = 72 vs. 18% *n* = 36; *p* = 0.002). No significant differences were detected in the rate of epidural anaesthesia or other pain reliever use. CS rate (27% *n* = 54 vs. 20% *n* = 41, *p* = 0.58) and vaginal-operative deliveries (15.5% *n* = 31 vs. 15.5% *n* = 31, *p* = 0.77) were not significantly different between the groups. Postpartum haemorrhage occurrence was also similar in both groups (12.9% *n* = 25 vs. 9% *n* = 18, *p* = 0.33). No uterine rupture occurred. Neonatal outcomes (Apgar score, cord blood pH, transfer to neonatal intensive care unit) were not significantly different in both groups. Women in the MVI group had a significantly shorter length of hospital stay calculated in hours as compared with women in the MVT group (MVI 97.63 ± 32. vs MVT 118.5 ± 123; *p* < 0.001). Further deliveries outcomes are summarized in Table 2.

**Table 1:** Patients characteristics

	MVI n 200	MVT n 200	*p*-Value
Median Age (range)	32 (28-35)	32(28-36)	> 0.05
Mean Parity (±SD)	0.5 ± 0.7	0.6 ± 1	> 0.05
Median BMI (range)	22.8 (20.7-25.3)	24.2 (21.6-27.6)	< 0.001
Mean week of gestational age at delivery(±SD)	40 ± 1	39 ± 1	> 0.05
Ethnicity (%)			
Europe	149 (74.5)	153 (76.5)	> 0.05
Africa	27 (13.5)	25 (12.5)	> 0.05
America	5 (2.5)	4 (2.0)	> 0.05
Asia	17 (8.5)	18 (9.0)	> 0.05
Missing	2 (1.0)	0 (0.0)	> 0.05
Premature rupture of membrane (%)	19 (9.5)	12 (6)	> 0.05
Median Bishop’s score (range)	2 (1-3)	2 (1-3)	1

Student’s t-test was used for Age, parity, BMI, week of gestational age, and Bishop score; chi-square test was used for Etnicity and Premature rupture of Membrane

*Abbreviations*: *BMI* Body mass index, *MVI* Misoprostol vaginal insert, *MVT* Misoprostol vaginal tablets

**Table 2:** MVI vs MVT deliveries outcomes

	MVI *n* = 200	MVT *n* = 200	*P* value
Vaginal delivery within 24 h n°(%)	127 (63.5%)	110 (55%)	< 0.001
Induction to vaginal delivery interval minutes mean (±SD)	1048 ± 814	1510 ± 1043	< 0.001
Predelivery oxytocin n°(%)	6 (3)	11 (5.5)	> 0.05
Peridural anesthesia n°(%)	79 (39.5)	65 (32)	> 0.05
Pain reliviers (excl. PDA) n°(%)	132 (66)	143 (71.5)	> 0.05
Uterine Hyperstimulation n°(%)	13 (6.5)	13 (6.5)	> 0.05
Tachysistole n°(%)	72 (36)	36 (18)	0.002
Apgar < 7 n (%)	8 (4)	2 (1)	> 0.05
pH art < 7.15 n° (%)	36/149 (24)	25/161 (15)	> 0.05
Umbelical artery base excess mean ± SD	−3.7 ± 3	−4.1 ± 3	> 0.05
Neonatal birth weight mean ± SD	3428 ± 429	3389 ± 471	> 0.05
Meconium n°(%)	29 (14)	29 (14)	> 0.05
NICU n°(%)	14 (7)	20 (10)	> 0.05

*Abbreviations: NICU* Neonatal intensive care unit, *MVI* Misoprostol vaginal insert, *MVT* Misoprostol vaginal tablets

Since uterine tachysistole was significantly higher in MVI group, the predictors of uterine tachysystole among patients induced with MVI were interrogated and no significant association were found with the demographic and clinical parameters and therefore predictive power is lacking (Table 3). Bishop’s score and parity were the strongest predictors of delivery within 24 h after adjusting for confounders in the same group (OR 0.90, CI 95% 0.85-0.96 *p* < 0.001) (Table 4).

**Table 3:** Predictors of uterine tachysystole in MVI group

	Crude OR (95% CI)	*p*-Values	Adjusted OR (95% CI)	*p*-Values
Age of mother	0.71 (0.42-1.20)	0.19	0.69 (0.38-1.26)	0.23
BMI	0.99 (0.93-1.06)	0.81	0.99 (0.92-1.06)	0.73
Ethnicity		0.82		0.87
Europe	1.00 (reference)		1.00 (reference)	
Africa	1.49 (0.65-3.42)		1.45 (0.56-3.72)	
America	1.24 (0.20-7.68)		1.35 (0.17-8.84)	
Asia	1.02 (0.36-2.91)		1.03 (0.30-3.23)	
Parity	1.05 (0.72-1.54)	0.80	1.06 (0.68-1.65)	0.79
Gestations age	1.05 (0.84-1.31)	0.65	1.05 (0.77-1.44)	0.75
Bishop’s score	1.01 (0.83-1.23)	0.92	1.02 (0.82-1.27)	0.86
over due date	1.00 (reference)		1.00 (reference)	
gestational diabetes mellitus	1.33 (0.57-3.12)		1.65 (0.54- 5.06)	
other	0.88 (0.44-1.73)		1.24 (0.49 -3.16)	
Fetus weight	1.23 (0.63-2.42)	0.54	1.23 (0.56-2.74)	0.60

Odds ratios (OR) and 95% CI for the occurrence of uterine tachysystole. Predictors from crude and fully adjusted logistic regression models. Age of the mother is taken in decades, and fetus weight was entered in kg

**Table 4:** Predictors of vaginal deliveries within 24 h in MVI group

	Crude OR (95% CI)	*p*-Values	Adjusted OR (95% CI)	*p*-Values
Age of mother	0.97(0.83-1.12)	0.64	1.02 (0.87-1.20)	0.81
BMI	1.02 (1-1.04)	0.41	1.01 (0.99-1.03)	0.25
Ethnicity		0.72		0.51
Europe	1.00 (reference)		1.00 (reference)	
Africa	0.9 (0.7-1.15)		0.96 (0.73-1.23)	
America	1.06 (0.61-1.82)		1.12 (0.66-1.88)	
Asia	1.10 (0.81-1.50)		1.23 (0.90-1.69)	
Parity	0.81 (0.73-0.90)	< 0.001	0.84 (0.74-0.95)	0.004
Gestations age	0.95 (0.89-1.01)	0.12	1.02 (0.94-1.11)	0.66
Bishop’s score	0.89 (0.84-0.94)	< 0.001	0.90 (0.85-0.96)	< 0.001
over due date	1.00 (reference)		1.00 (reference)	
gestational diabetes mellitus	1.37 (1.06-1.75)		1.43 (1.05-1.94)	0.55
other	1.22 (1-1.48)		1.14 (0.92 -1.41)	061
Fetus weight	0.91(0.75-1.11)	0.34	1.23 (0.56-2.74)	0.22

Odds ratios (OR) and 95% CI for the occurrence of vaginal delivery within 24 h. Predictors from crude and fully adjusted logistic regression models. Age of the mother is taken in decades, and fetus weight was entered in kg

## Discussion

MVT was compared to other methods such as oxytocin, Dinoprostone and placebo in several clinical randomized trials in terms of efficacy (vaginal delivery within 24 h) and safety (perinatal or maternal outcomes) (8). Recently, MVI was compared to Dinoprostone vaginal insert, a prostaglandin E2 analog in a phase III trial reporting significantly reduced times to delivery (efficacy) and no evidence of differences in maternal or neonatal safety outcomes (safety) (17). Since this study, three studies have compared the vaginal insert to other induction methods in terms of delivery outcomes [19–21]. However, none of these studies have compared MVI to MVT.

Our study shows for the first time that labour induction with MVI has a significant higher rate of vaginal delivery within 24 h, and a shorter hospital stay compared to the labour induction with MVT. The two groups of patients were homogeneous for clinical and demographic characteristics, except for the BMI (*p* < 0.001). However, mean BMI values were lower than 25.00 Kg/m2 both in MVI and MVT groups. Since the overweight women with a BMI up to 25.00 kg/m^2^are at increased risk of pregnancy complications at birth [22], we can speculatively justify the similar number of operative deliveries and CSs, and neonatal outcomes in the two groups despite the difference in BMI.

Furthermore, in MVI an increased incidence of uterine tachysystole was reported. However, this finding was not accompanied by an higher rate of operative deliveries, CS; similarly, neonatal outcomes do not differ among the groups. Interesting, we found that uterine tachysystole cannot be predicted by demographic or clinical factors.

In our study, adverse events such as CS, postpartum haemorrhage, meconium-stained amniotic fluid, Apgar score below 7, fetal acidosis (defined by arterial pH < 7.15), and neonatal complications were similar in both groups. In line with previous studies, we detected a relatively high incidence of uterine tachysystole after Misoprostol use [8, 23, 24]. For example, Jozwiak et al. reported a 61% reduction of uterine hyperstimulation in the Foley catheter group compared to the use of 25 μg MVT and Hofmeyr et al. reported a lower incidence of tachysystole with similar results using vaginal / intracervical dinoprostone [8, 23]. Efficacy of misoprostol seems to be correlated with the dosage and therefore several studies focused on determining the optimal dosing regimen. In our study the dosages used in the MVT group was previously reported as the most effective for vaginal delivery success and is associated with the lowest rates of tachysystole [8]. Similarly, the efficacy and safety of MVI was evaluated with different dosages. For example, Wing et al. compared three different doses of MVI and determined that 200 μg was the most effective for the onset of active labour within 24-h but with the disadvantage of an increased rate of tachysystole (41.2%) compared to MVI 150 μg (25.6%) and MVI 100 μg (19.5%) [25]. In our study, the incidence of tachysytole was significantly higher than in the MVT group (36% vs. 18%). However, this higher incidence did not result in adverse maternal and/or neonatal outcomes. Further, the multivariate analysis displayed no maternal or fetal predictive factors for tachysytole. We hypothesize that the main reason for the higher incidence of uterine tachysytole are the pharmacokinetic properties of MVI such as the relatively long elimination half-time after removal of 40 min [16]. Thus, timely removal of the vaginal insert may reduce the incidence of tachysytole. Whether the inclusion of currently excluded patients with certain characteristics such as multiparty, low BMI, or rupture of membranes is warranted, needs to be evaluated in further prospective studies.

A general goal of clinical management during pregnancy is to avoid adverse maternal and fetal outcomes while avoiding unnecessary CS. For this reason, ACOG recommend in uneventful pregnancies inductions of labour at 41 0/7 weeks of gestations to reduce the number of elective CS and to improve perinatal outcomes [15]. However, several studies have shown an increased rate of failed induction and CS if women are induced with an unfavourable cervix [4–7]. In this context prostaglandins are effective agents for cervical ripening [8]. In our study, we observed that MVI did not reduce the CS rate but the time to delivery and therefore a significant reduction of hospital stay. Theoretically, the shorter hospital stay may be related to the higher rate of vaginal delivery within 24 h, however it is just a speculative assumption. If so, it might be beneficial for patients, particularly for those needing a rapid delivery such as late onset preeclampsia [26]. Although MVI (Misodel, 74.37 USD) might be more expensive compared to the MVT (Cytotec tablet, 0.26 USD), the reduced hospital stay outweighs this disadvantage. Furthermore, the litigation risks inherent to the use of misoprostol off-label can be avoided using the approved MVI preparation. A further important aspect is the women’s preference regarding comfort and pain during induction of labour. As reported by Impey et al., women’s expectation is to have a *safe and fast* labour with little pain [27]. Another study showed that 40% of women who experienced a labour induction expressed the desire to minimize the time duration of labour induction in case of the necessity of labour induction in the following pregnancy [28]. In this regard, MVI has the potential to increase patient satisfaction by decreasing the time to delivery interval. Limit of this study is its retrospective nature, and prospective future studies are needed to confirm our proof of concept.

## Conclusions

MVI is able to induce higher rate of vaginal delivery within 24 h compared to MVT. A shorter hospital stay was also reported in the MVI group. The higher rate of tachysystole after MVI induction may represent the price to be paid for a quicklier time interval from induction to delivery. However, although more tachysystole events are reported after MVI induction, no differences in maternal and neonatal outcomes as well as in operative deliveries and CS are observed when comparing to MVT. Furthermore, no predictors of vaginal delivery within 24 h are identified in the MVI group, except for Bishop score and parity. It means that probably we need further prospective studies in order to identify which modifiable predictors can be used in the future to better select the patients to a more appropriate labour induction program.

## Abbreviations

BMI: Body mass index
CS: Caesarean sections
IDI: Induction-to-vaginal delivery interval
MVI: Misoprostol vaginal insert
MVT: Misoprostol vaginal tablets
